# Chikungunya virus infection in the skin: histopathology and cutaneous immunological response

**DOI:** 10.3389/fmicb.2025.1497354

**Published:** 2025-01-28

**Authors:** Natália Gedeão Salomão, Luciana Araújo, Luiz José de Souza, Anna Luiza Young, Carlos Basílio-de-Oliveira, Rodrigo Panno Basílio-de-Oliveira, Jorge José de Carvalho, Priscilla Conrado Guerra Nunes, Juliana Fernandes da Silva Amorim, Douglas Valiati dos Santos Barbosa, Marciano Viana Paes, Kíssila Rabelo, Flavia Dos Santos

**Affiliations:** ^1^Laboratório das Interações Vírus-Hospedeiros, Instituto Oswaldo Cruz/Fundação Oswaldo Cruz (IOC/Fiocruz), Rio de Janeiro, Brazil; ^2^Laboratório Interdisciplinar de Pesquisas Médicas, Instituto Oswaldo Cruz/Fundação Oswaldo Cruz (IOC/Fiocruz), Rio de Janeiro, Brazil; ^3^Departamento de Anatomia Patológica, Universidade Federal do Estado do Rio de Janeiro (UNIRIO), Rio de Janeiro, Brazil; ^4^Faculdade de Medicina de Campos, Campos dos Goytacazes, Brazil; ^5^Laboratório de Ultraestrutura e Biologia Tecidual, Universidade do Estado do Rio de Janeiro (UERJ), Rio de Janeiro, Brazil; ^6^Laboratório de Análise Imunomolecular, Instituto de Tecnologia em Imunobiológicos (Bio-Manguinhos/Fiocruz), Rio de Janeiro, Brazil

**Keywords:** chikungunya virus, histopathology, skin, immunohistochemistry, immune response

## Abstract

*Alphavirus chikungunya* virus (CHIKV) is an arbovirus, belonging to the *Togaviridae* family. The disease caused by CHIKV generally evolves with spontaneous resolution in a few weeks; however, progression to a chronic disease may occur. The most common symptoms are fever, myalgia, and arthralgia; however, skin manifestations may occur in 40 to 80% of infected individuals. Morbilliform and maculopapular erythematous eruptions, vesiculobullous lesions, generalized erythema, maculopapular eruption and skin peeling, hypermelanosis, painful oral lesions, and urticarial lesions have been reported. Usually, these manifestations disappear, but they can become sequelae. Since the skin is the first line of defense against CHIKV infection, in this study, we aimed to investigate the immunohistopathological aspects of the skin of infected individuals during the acute phase of the disease by performing histopathological and ultrastructural analysis, detection and quantification of the viral genome, detection of viral antigen and immune cells, and cytokines/chemokines’ characterization. The main histopathological findings were perivascular and inflammatory infiltrates, blood capillary ectasia, and interstitial edema. The immunohistochemistry revealed CHIKV antigen in the epidermis, endothelial cells, fibroblasts, and macrophages in the reticular and papillary dermis; inflammatory cells infiltrate; arrector pili muscle; sweat and sebaceous glands; and hair follicle. Moreover, inflammatory infiltrates were composed of lymphocytes (CD4^+^ and CD8^+^) and macrophages (CD68^+^) in the dermis and perivascular infiltrate. TNF-*α*, IL-6, RANTES, and VEGFR2 were expressed in the epidermis, blood vessels, sweat glands, and migrating cells. Loss of contact among adjacent keratinocytes, epidermis presenting necrotic cells, and fibroblasts with dilated cisternae in the endoplasmic reticulum and mitochondria with few cristae was observed by transmission electron microscopy. Studies involving skin immunopathogenesis during CHIKV infection are still scarce; therefore, the findings presented here can contribute to a better understanding of the disease immunopathogenesis.

## 1 Introduction

*Alphavirus chikungunya* virus (CHIKV) is an arbovirus belonging to the *Togaviridae* family, mainly transmitted through the bite of infected female *Aedes* mosquitoes, although vertical transmission and blood transfusion infections have also been reported. In general, Chikungunya fever evolves to a spontaneous resolution within a few weeks; however, in some cases, evolution to a chronic and severe disease that can last months or years may occur (Gasque et al., 2016; Ojeda Rodriguez et al., 2023). In the acute phase of the disease, the most common symptoms are fever, polyarthralgia, and rash, but headache, fatigue, rash, nausea, vomiting, conjunctivitis, and myalgia may occur (Simon et al., 2008). As dermal changes, morbilliform and maculopapular eruptions of erythematous lesions on the trunk, face, and extremities were quite common findings, with occurrence in 20 to 80% of those infected (Bandyopadhyay and Ghosh, 2010; Kumar et al., 2017; Rueda et al., 2018; Spoto et al., 2018; Tini and Rezza, 2018). The rash can be extensive, affecting more than 90% of the skin (Burt et al., 2017). Vesiculobullous lesions may occur in adults; however, they are more common in older patients (Robin et al., 2010; Singh et al., 2014; Farias et al., 2019), including those cases of vertical transmission (Torres et al., 2016; Jebain et al., 2020). In infants, generalized erythema, maculopapular rash, and peeling of the skin are quite observed (Valamparampil et al., 2009). In addition to these, hypermelanosis, oral lesions with the occurrence of pain and urticarial lesions, severe purpuric lesions, and necrosis in the nasal region have already been reported (Bandyopadhyay and Ghosh, 2010; Casais et al., 2020; Sharif et al., 2021). Mostly, these cutaneous manifestations disappear; however, in some cases, they can reappear weeks after the acute phase and persist for months. Moreover, those can even become sequelae, such as reversible post-inflammatory hypopigmentation (Bandyopadhyay and Ghosh, 2010; Kumar et al., 2017). Regular application of emollients is necessary to relieve the symptoms (Bandyopadhyay and Ghosh, 2010). Some patients who became infected with CHIKV and were slightly affected in the long term by the disease reported a significantly higher prevalence of skin diseases (Doran et al., 2022). It appears that CHIKV infection can exacerbate existing dermatoses, such as psoriasis, and favor the unmasking of undiagnosed leprosy (Inamadar et al., 2008). Epidermolysis bullosa is considered a complication of CHIKV infection by the Brazilian Ministry of Health, which can lead to death (Ministério-da-Saúde, 2017). Therefore, this study aimed to investigate the histopathological and immunological aspects of acute CHIKV infection in the skin of nine patients with cutaneous manifestation during an epidemic in Campos dos Goytacazes, Rio de Janeiro (RJ), Brazil, in 2019.

## 2 Materials and methods

### 2.1 Ethical statement

The study was approved by the Ethics Committee of the Oswaldo Cruz Foundation (CAEE: 92728218.5.0000.5248). All the patients authorized the publication of the results, by providing written informed consent.

### 2.2 Case descriptions

The skin of nine patients with cutaneous manifestations during the Chikungunya epidemic that occurred in Campos dos Goytacazes, Rio de Janeiro (RJ), in 2019, was selected for investigation. Patients were assisted at the Regional Center for Infectious Diseases of the Plantadores de Cana Hospital. In that year, the disease incidence was 374.05 cases per 100,000 inhabitants in RJ (CGVS, 2019). Some characteristics of the study population are described below, and the frequencies are shown in Supplementary Table S1.

- Case 1: Female, without comorbidity reported and 27 years old, presenting symptoms such as headache and fever. The biopsy was performed 1 day after the onset of symptoms. This patient required hospitalization and had negative results for bacterial infection.
- Case 2: Female, hypertensive, and 59 years old reporting anorexia, arthralgia, headache, diarrhea, rash, fever, pruritus, dysgeusia, nausea, and lymphadenomegaly. Biopsy was performed 8 days after the onset of symptoms. This patient required hospitalization and had negative results for bacterial infection.
- Case 3: Pregnant, 32 years old, reporting symptoms such as fever, headache, nausea, arthralgia, edema, rash, pruritus, and lymphedema. The biopsy was performed 10 days after the onset of symptoms. This patient required hospitalization and had negative results for bacterial infection.
- Case 4: Female, hypertensive, and 52 years old reporting arthralgia, diarrhea, rash, fever, pruritus, vomiting, dysgeusia, and edema. Biopsy was performed 9 days after the onset of symptoms.
- Case 5: Female, age unavailable, without comorbidity reported, presenting headache, arthralgia, and myalgia.
- Case 6: Female, without comorbidity reported, and 47 years old, reporting headache, polyarthralgia, and fever. The biopsy was performed 12 days after the onset of symptoms.
- Case 7: Male, without comorbidity reported, 26 years old presenting symptoms such as arthralgia, anorexia, diarrhea, rash, fever, pruritus, vomiting, dysgeusia, and paresthesia. Biopsy was performed 7 days after the onset of symptoms.
- Case 8: Female, without comorbidity reported, 50 years old presenting anorexia, arthralgia, diarrhea, rash, fever, pruritus, vomiting, dysgeusia, paresthesia, edema, and nausea. Biopsy was performed 7 days after the onset of symptoms.
- Case 9: Female hypertensive, osteoarthritis, and depression, 59 years old reporting myalgia, headache, arthralgia, and fever. The biopsy was performed 10 days after the onset of symptoms.

### 2.3 Sample collection

Patients with skin manifestations and a positive anti-CHIKV IgM result by lateral flow immunochromatographic test performed at the health unit had blood collected for confirmation by serological testing. Skin biopsies (6 mm punch) were collected at the site of the lesion or skin manifestation depending on each patient. The skin samples were fixed in 10% buffered formalin and glutaraldehyde and also stored at −20°C without fixation. Health professionals at Plantadores de Cana Hospital, located in Campos dos Goytacazes, Rio de Janeiro (RJ), were responsible for patient care and serological diagnosis. Three samples of skin from healthy donors undergoing bariatric surgery were selected as controls.

### 2.4 Serological diagnosis

The anti-CHIKV IgM ELISA kit (Euroimmun, Lubeck, Germany) was used to detect specific anti-CHIKV IgM antibodies according to the manufacturer’s instructions.

### 2.5 Viral RNA extraction and RT-qPCR for chikungunya virus detection in skin samples

The PureLink™ FFPE RNA Isolation Kit (Invitrogen, Carlsbad, California, USA) was used to extract RNA from formalin-fixed paraffin-embedded (FFPE) skin samples, following the manufacturer’s instructions. RNA extraction from frozen skin samples was performed by using the IndiSpin® Pathogen Kit (Indical Bioscience, Leipzig, Germany), and CHIKV RNA was detected by using the RT-qPCR one-step Kit TaqMan Fast Virus 1 Step (Applied Biosystems, Foster City, California, USA), according to the protocol described by Lanciotti et al. (2007), using the ABI 7500 System (Applied Biosystems, Foster City, California, USA).

### 2.6 Histopathological analysis

Skin samples fixed in 10% buffered formalin were submitted to an automatic tissue histological processor, where samples were briefly dehydrated in ethanol, clarified in xylene, and impregnated with paraffin. Next, paraffin embedding was performed to create paraffin blocks, containing the skin sample, and further sectioned using a microtome into 5 μm sections on glass histological slides. To perform histopathological analysis, the slides with tissue sections were incubated for 90 min at 60°C for paraffin melting. After incubation, skin tissues were deparaffinized in xylene and rehydrated with decreasing concentrations of ethanol (100 to 70%) up to the addition of water. The slides were subsequently stained with hematoxylin and eosin (H.E.) and visualized by light microscopy (Olympus BX 53F, Japan). Digital images of histological features were obtained using a coupled Olympus DP72 camera and Image-Pro Plus software version 4.5. All pictures were assembled using Adobe Photoshop CS6.

### 2.7 Immunohistochemistry analysis

For immunohistochemistry, the paraffin-embedded tissues were cut in 4 μm thick. Deparaffinization and rehydration were performed as previously described. Aiming to re-expose the antigenic sites, masked due to fixation with formalin, antigen retrieval was performed by heating the tissue in citrate buffer solution (pH 6.0). Blocking endogenous peroxidase was performed with 3% hydrogen peroxide in methanol for 10 min and washing steps with Tris–HCl (pH 7.4). Protein Blocker solution (ScyTek, Logan, Utah, USA) was used in skin tissues for 10 min to reduce non-specific binding. After blocking, tissue samples were incubated overnight at 4°C with a polyclonal anti-CHIKV mouse hyperimmune ascites fluid, diluted 1:700 (kindly provided by Dr. Livia Martins, from the Section of Arbovirology and Hemorrhagic Fevers, Instituto Evandro Chagas, Pará); and anti-human monoclonal antibodies against CD4 (Spring Bioscience, Pleasanton, CA, USA, diluted 1:100), CD8 (Santa Cruz Biotechnology, Dallas, Texas, USA, diluted 1:100), and CD68 (Santa Cruz Biotechnology, Dallas, Texas, USA, diluted 1:100); TNF-*α* (Santa Cruz Biotechnology, Dallas, Texas, USA, diluted 1:100); IL-6 (Santa Cruz Biotechnology, Dallas, Texas, USA, diluted 1:100); RANTES (Abcam, Cambridge, United Kingdom, diluted 1:200); and VEGFR2 (Spring Bioscience, Pleasanton, CA, USA, diluted 1:200). On the next day, tissue samples were incubated with two-step polymer immunohistoprobe Plus (Redwood, California, USA) and amplifier for mouse and rabbit IgG for 15 min, and HRP polymer detector at room temperature for 15 min. After incubation, diaminobenzidine (ScyTek, Logan, Utah, USA) was added. Counterstaining was performed with Harris’ hematoxylin solution (Dako, Palo Alto, California, USA), and slide visualization was performed using light microscopy (Olympus BX 53F, Japan). The digital images of the detections carried out were taken using a coupled Olympus DP72 camera and the Image-Pro Plus software version 7.

### 2.8 Quantification analysis

For quantification analysis for each antibody, images from 20 random fields (controls and cases) were acquired in an Olympus BX 53F microscope with a coupled Olympus DP72 camera, using the software Image Pro version 7. For CD4, CD8, and CD68, the acquisition was at 400x magnification, and the result was expressed in the number of positive cells/random fields. For TNF-*α*, IL-6, RANTES, and VEGFR2, at 1000x magnification, the result was expressed in % of the positive area, using Image J software.

### 2.9 Electron microscopy analysis

The skin tissue samples of two cases were fixed with 2.5% glutaraldehyde in 0.1 M sodium cacodylate buffer (pH 7.2) and post-fixed with 1% buffered osmium tetroxide. The dehydration was performed using increasing concentrations of acetone (30, 50, 70, 90, and 100%) and embedded in EPON at 60°C for 3 days. Ultrathin sections (60 nm) were contrasted with uranyl acetate and lead citrate before visualization on a JEOL 1001 transmission electron microscope (Jeol Ltd., Tokyo, Japan).

### 2.10 Statistical analysis

Data were analyzed with GraphPad Prism software v 6.0 (GraphPad Software, San Diego, California, USA). The statistical difference between cases and controls was performed by an unpaired *t*-test with a significant level of 0.05 (*p* < 0.05).

## 3 Results

### 3.1 Cutaneous manifestations in the acute phase of chikungunya infection

**Figure 1:**
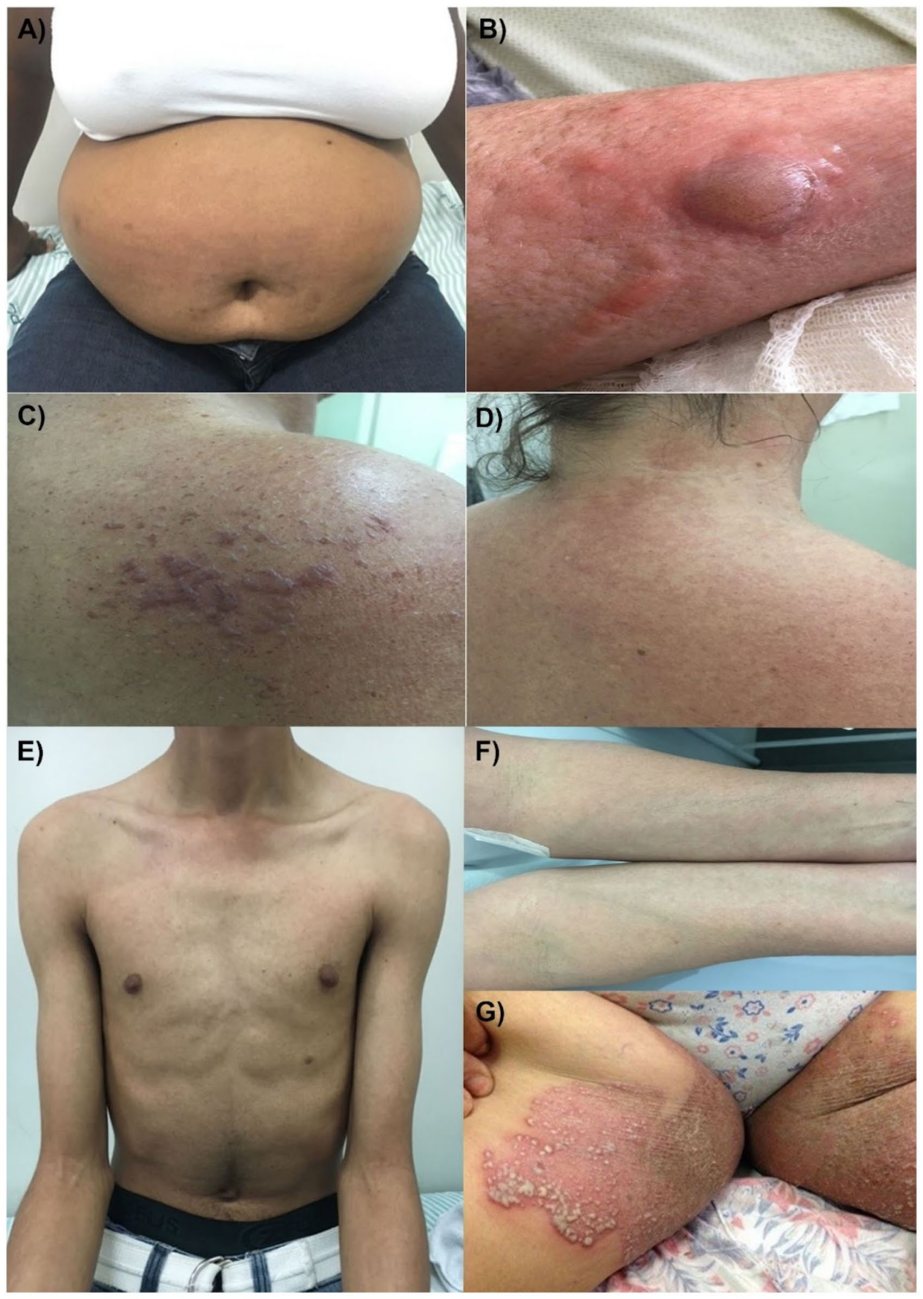
Cutaneous clinical manifestations of patients during the acute phase of CHIKV infections: **(A)** Case 2: urticaria; **(B)** Case 3: vesiculobullous lesion; **(C)** Case 4: minor vesiculobullous lesions; **(D)** Case 4: erythema; **(E)** Case 7; and **(F)** Case 8: diffuse papular erythematous rash; and **(G)** Case 9: vesiculopustular lesions, erythema, and scaling.

Cutaneous manifestations observed included urticated plaques in the abdominal region (Figure 1A), vesiculobullous lesions in the right lower limb (Figure 1B), symmetric vesiculobullous lesions sometimes content hemorrhagic or crystalline (Figure 1C), erythema on the back of the neck (Figure 1D), papular erythematous rash (Figures 1E,F), and vesicopustular lesions on an erythematous and scaly base (Figure 1G). The clinical cutaneous manifestations are described in Supplementary Table S2.

### 3.2 The skin of patients with chikungunya in the acute phase showed histopathological changes

**Figure 2:**
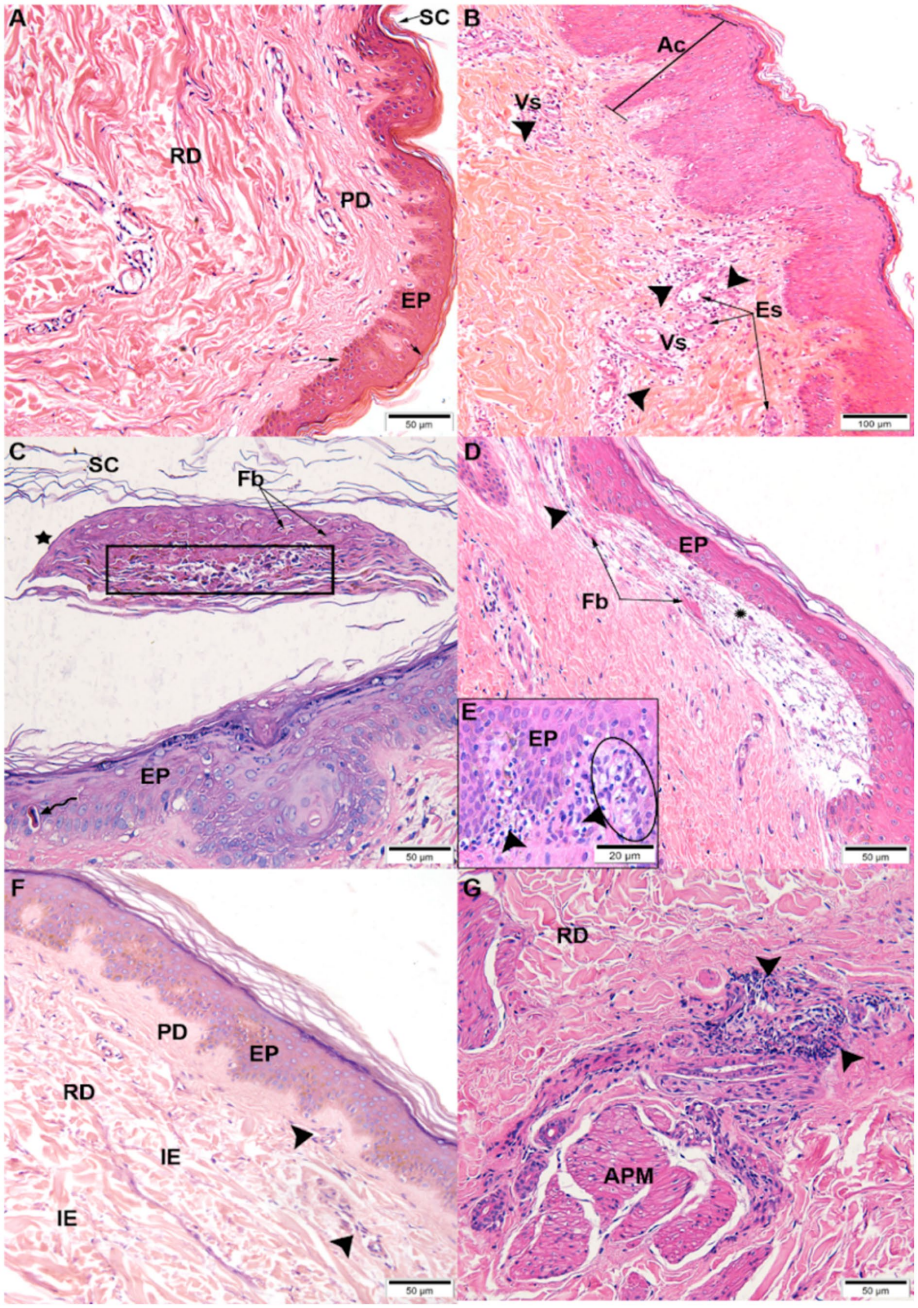
Histopathological changes observed by hematoxylin and eosin staining: **(A)** Control, non-infected skin with regular appearance, stratum corneum (SC), epidermis (EP), papillary dermis (PD), and reticular dermis (RD) | CHIKV-infected skins: **(B)** acanthosis (Ac), perivascular infiltrate (➤), and endothelial swelling (Es) of blood vessels (Vs); **(C)** subcorneal intraepidermal blister (⋆) containing cellular debris (rectangle) and fibrin (Fb). Epidermis (EP) with apoptotic keratinocyte (⇜); **(D)** subepidermal blister (⁕) containing inflammatory infiltrate (➤) and fibrin (Fb); **(E)** inflammatory infiltrate (➤) in the papillary dermis (PD) and basal vacuolation (circle); **(F)** interstitial edema (IE), inflammatory infiltrate (➤) in the papillary (PD) and reticular dermis (RD); **(G)** inflammatory infiltrate (➤) near the arrector pili muscle (APM).

The nine samples positive for CHIKV antigen by immunohistochemistry were analyzed for histopathological changes. In the control skin from a non-infected CHIKV patient, no changes in the dermis (papillary and reticular) and epidermis (basal, spinous, granulosa, and corneal layer) were observed (Figure 2A). However, the skin from patients presenting acute CHIKV infection showed acanthosis, characterized by thickening of the epidermis, perivascular infiltrate, and endothelial swelling (Figure 2B); appearance of a blister in the epidermal layer and below the corneal layer (subcorneal intraepidermal) with content of cellular debris and fibrin; in addition to the epidermis with the presence of keratinocytes with eosinophilic cytoplasm, characteristic of apoptosis (Figure 2C); blister below the epidermis (subepidermal) containing inflammatory infiltrate and light fibrin (Figure 2D); contact dermatitis characterized by an inflammatory infiltrate in the region of the papillary dermis going toward the epidermis, in addition to the vacuolization of the keratinocyte cytoplasm in the basal layer, called basal vacuolization (Figure 2E); presence of interstitial edema between the collagen fibers of the reticular dermis, inflammatory infiltrate in the papillary and reticular dermis (Figure 2F); inflammatory infiltrate close to the erector pilosis muscle (Figure 2G). The histopathological findings are described in Supplementary Table S3.

### 3.3 CHIKV viral RNA and antigen were detected in skin biopsies

To detect the CHIKV viral genome, frozen (*n* = 3) and paraffin-embedded (*n* = 3) skin samples were submitted to viral RNA extraction and analyzed by RT-qPCR for molecular detection. Two of the three frozen skins were positive (2/3; 66.7%), while the three paraffin-fixed skins were positive for CHIKV (3/3: 100%) (Table 1).

**Table 1:** CHIKV RNA detection in skin biopsies by RT-qPCR.

Case	Material	RT-qPCR	Average copies/mL
1	Paraffin	Positive	1.13E+04
2	Frozen	Positive	3.90E+03
3	Paraffin	Positive	8.54E+02
4	Frozen	Positive	2.28E+04
5	Paraffin	Positive	3.56E+03
6	Frozen	Negative	-

**Figure 3:**
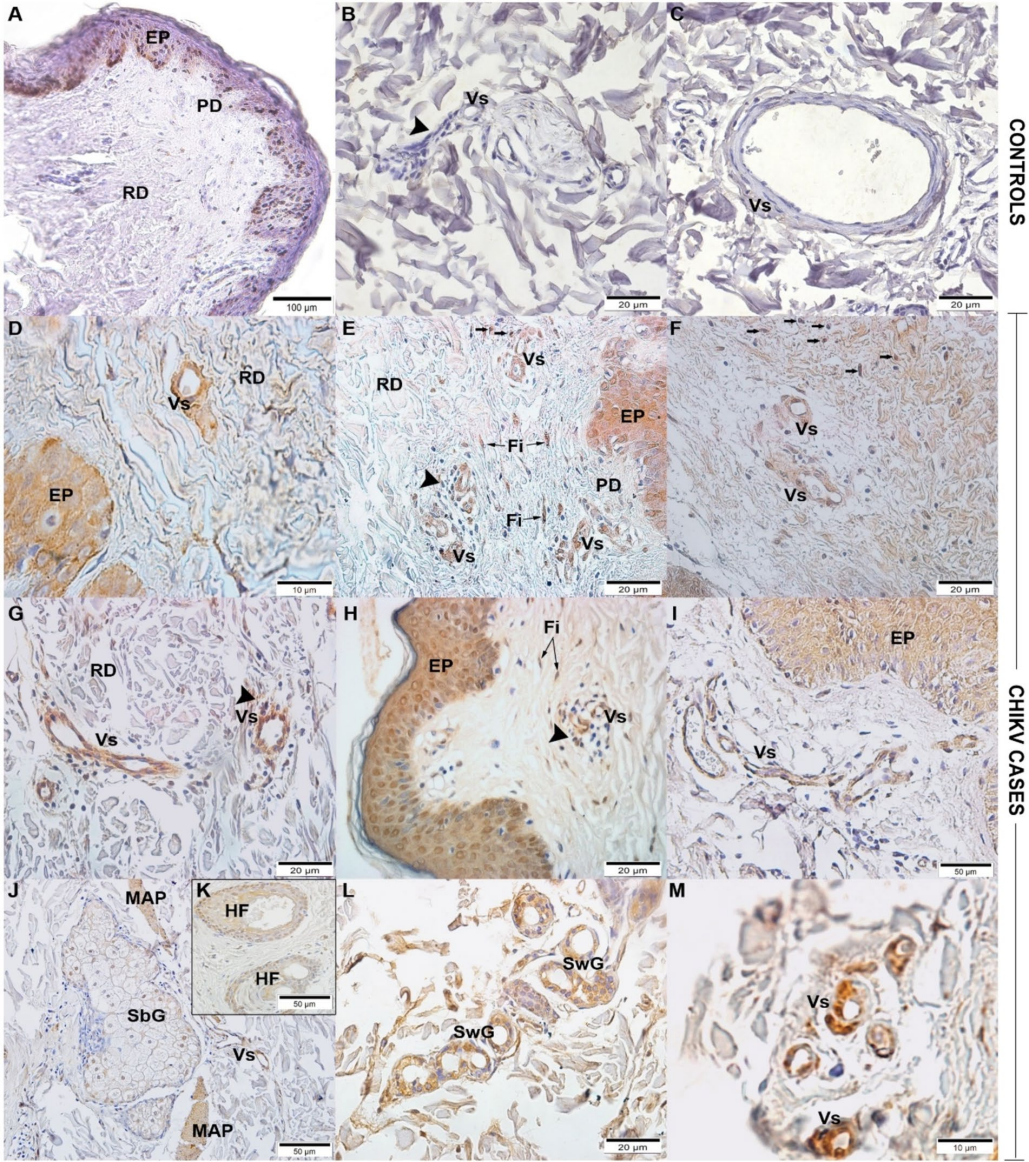
Detection of CHIKV viral antigen in skin biopsy by immunohistochemistry: Control skin without detection of CHIKV antigen **(A–C)**. Skins with CHIKV antigen detection in: **(D)** Case 1 - epidermis (EP) and blood vessels (Vs) in the reticular dermis (RD); **(E)** Case 2 - epidermis (EP) and blood vessels (Vs) in the papillary (PD) and reticular dermis (RD), perivascular infiltrates and fibroblasts (Fi), and other cells (➞) running through the dermis; **(F)** Case 3 - blood vessels (Vs) and cells (➞) running through the dermis; **(G)** Case 4 - blood vessels (Vs) in the reticular dermis (RD); **(H)** Case 6 - epidermis (EP) and blood vessels (Vs), perivascular inflammatory infiltrate (➤), and fibroblasts (Fi); **(I)** Case 9 - epidermis (EP) and blood vessels (Vs); **(J,K)** Case 7 - sebaceous glands (SbG), arrector pili muscle (APM), blood vessels (Vs), and hair follicle (HF); **(L)** Case 8 - sweat glands (SwG); **(M)** ICS - blood vessels (Vs).

By immunohistochemistry, CHIKV antigen was detected mainly in the epidermis, endothelial cells of blood vessels of the reticular and papillary dermis, perivascular inflammatory infiltrates, cells in the dermis, with a morphology such as fibroblasts and macrophages, arrector pili muscle, and sweat and sebaceous glands, in addition to the hair follicle (Figures 3D–M). As expected, CHIKV antigen was not detected in control samples (Figures 3A–C).

### 3.4 Characterization of cell types in CHIKV-infected skin biopsies

**Figure 4:**
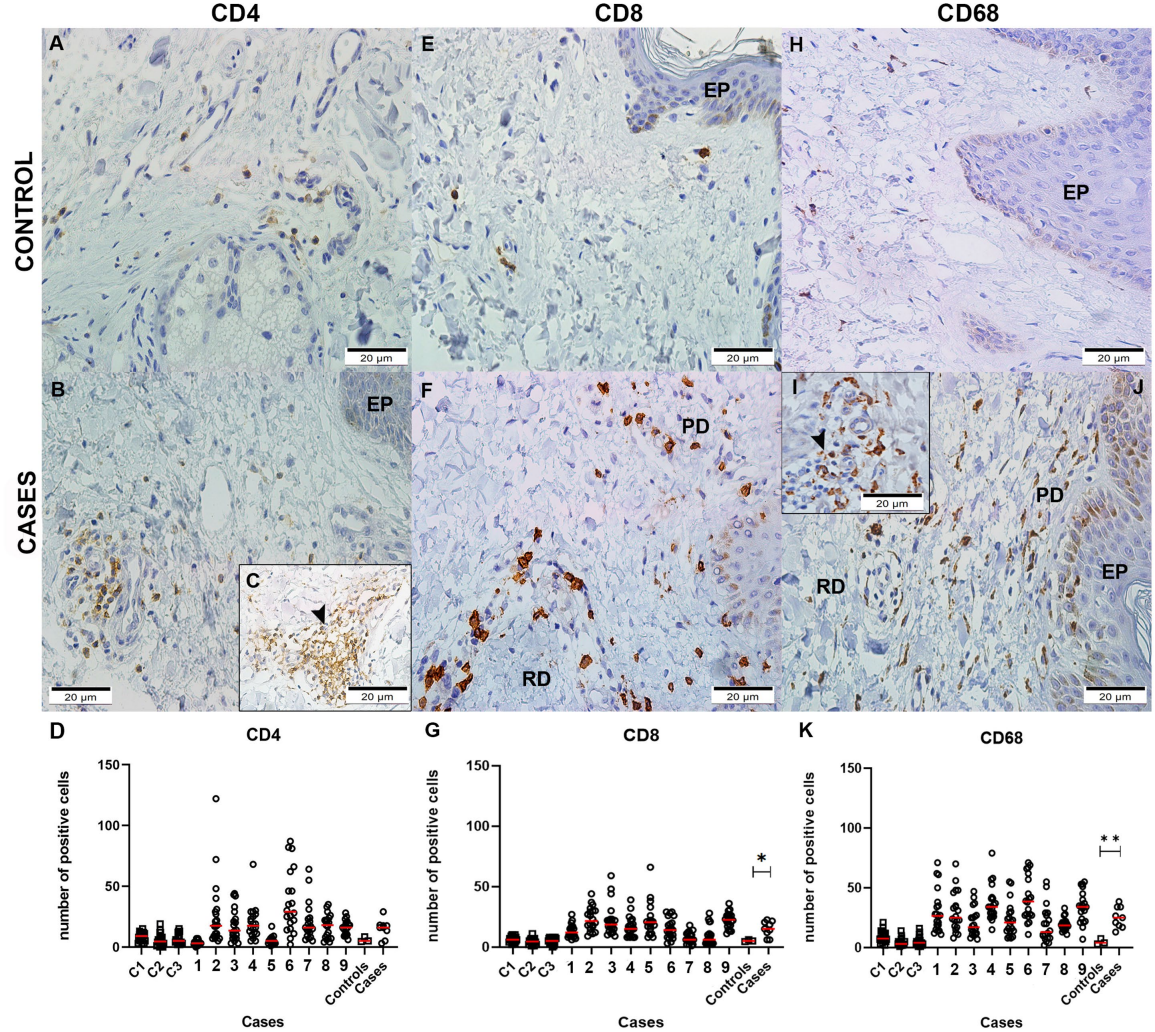
Detection of CD4+, CD8+, and CD68+ cells: Control skins with detection of **(A)** CD4^+^, **(E)** CD8^+^, and **(H)** CD68^+^ cells. Skins of patients infected with CHIKV: **(B,C)** CD4+ in blood vessels (Vs) in reticular dermis (RD) and in areas of inflammatory infiltrate (➤); **(F)** CD8+ in reticular and papillary dermis (PD); **(I)** CD68+ in blood vessels and **(J)** reticular and papillary dermis. Quantification of cells in controls and cases: **(D)** CD4; **(G)** CD8; and **(K)** CD68. (*p < 0.05).

The cell profile in the CHIKV-infected skin was characterized by immunohistochemistry using anti-CD4+, anti-CD8+, and CD68+ cells’ antibodies. Representative images of control (Figures 4A,E,H) and CHIKV-infected skin are shown below (Figures 4B,C,F,I,J). To confirm whether there was a difference in the number of positive cells between the control and the case of the following cells, quantification was performed (Figures 4D,G,K).

### 3.5 Cytokines and chemokines expressions in CHIKV-infected skin biopsies

**Figure 5:**
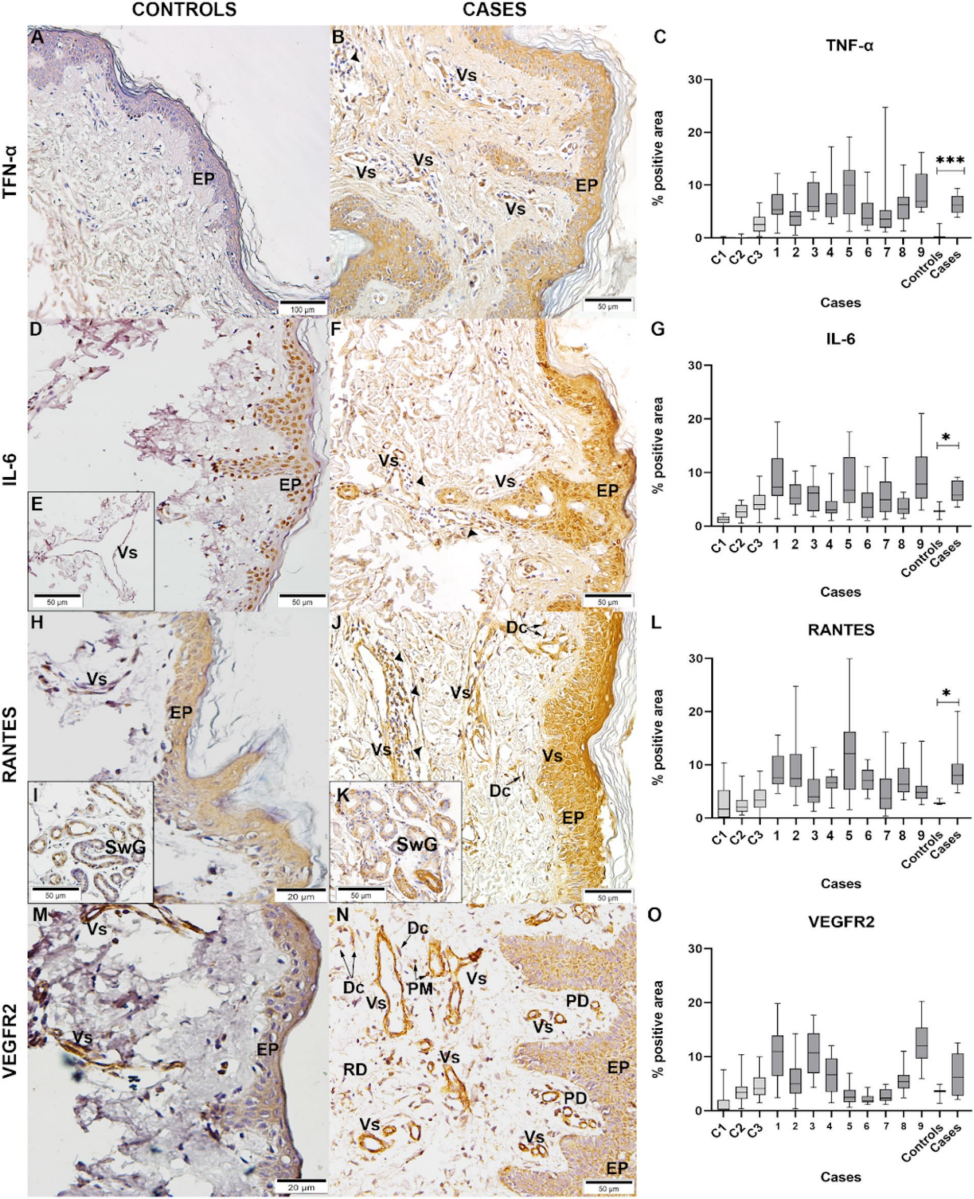
Detection of cytokines/mediators: Control skin: **(A)** TNF-*α*; **(D,E)** IL-6; **(H,I)** RANTES; **(M)** VEGFR2. CHIKV skins, with expression of **(B)** TNF-α, **(F)** IL-6, **(J,K)** RANTES, and **(N)** VEGFR2. In the epidermis (EP), blood vessels (Vs), inflammatory infiltrate (➤), sweat glands (SwG), dermal cells (Dc), reticular dermis (RD), dermal papilla (PD), polymorphonuclear (PM) cells. Percentual of positive areas expressing these cytokines/mediators: **(C)** TNF-α, **(G)** IL-6, **(L)** RANTES, and **(O)** VEGFR2 (*p < 0.05).

To describe the inflammatory response profile in CHIKV-infected skin biopsies, we evaluated the expression of pro-inflammatory cytokines, such as TNF-*α* and IL-6. The first one was expressed mainly in keratinocytes of the epidermis and endothelial cells of blood vessels (Figure 5B), while the latter was also expressed in sweat glands (Figure 5F). In control, IL-6 expression was more evident in the nuclei of epidermal cells (Figure 5D) and weakly in blood vessels (Figure 5E). RANTES is an important inflammatory mediator and plays a role in immune cells’ recruitment to the site of infection. Its expression was observed in the epidermis and blood vessels surrounded by many inflammatory cells (Figure 5J) and in sweat glands with swollen cells (Figure 5I) compared to the control (Figure 5H). VEGFR2—vascular endothelial growth factor receptor 2—was detected mainly in blood vessels and fibroblasts and less in the epidermis (Figure 5K). RANTES and VEGFR2 play a significant role in regulating vascular permeability. Control skins (Figures 5A,D,E,H,I,M) showed no or weak expression of the different markers. Statistically significant differences were observed in TNF-α (Figure 5C), IL-6 (Figure 5G), and RANTES (Figure 5L) expressions but not in VEGFR2 (Figure 5O).

### 3.6 Ultrastructural analysis of skin biopsies from CHIKV-infected patients

**Figure 6:**
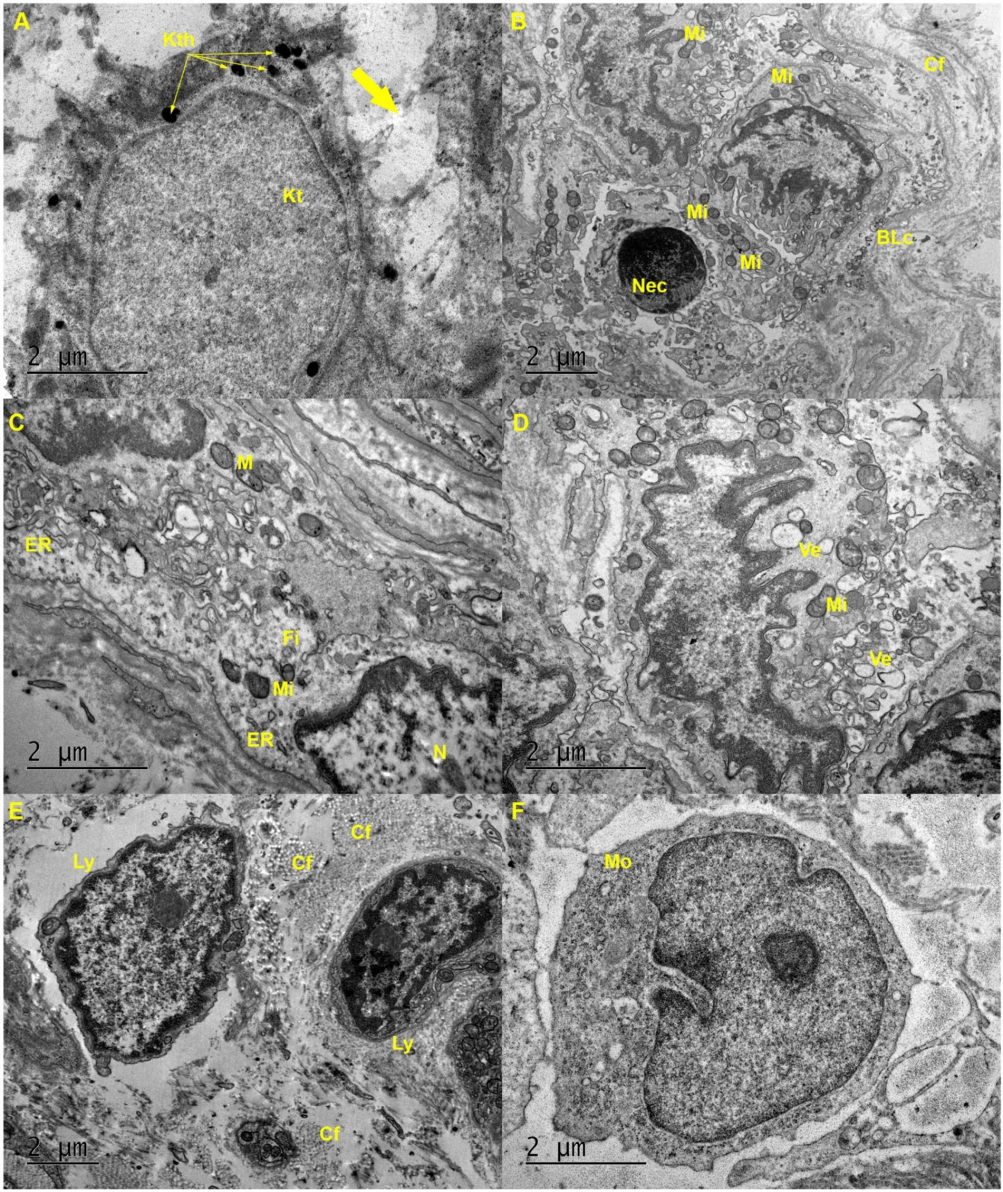
Ultrastructure of skin biopsies infected with CHIKV: **(A)** Keratinocyte (Kt) from the spinous layer of the epidermis exhibits keratohyalin (Kth) granules, but with loss of tonofilaments and desmosomes that make contact (thick arrow) with adjacent keratinocytes. **(B)** Epidermis with necrotic cells (Nec), basal layer cells (BLc) with mitochondria (Mi) in the absence of cristae, and collagen fibers (Cf) in the dermis. **(C)** Fibroblast (Fi) shows an endoplasmic reticulum (ER) with dilated cisternae and mitochondria (Mi) with few cristae. **(D)** Dendritic cells present mitochondria (Mi) with few cristae and production of vesicles (Ve). **(E)** Presence of lymphocytes (Ly) infiltrated in the dermis between collagen fibers (Cf). **(F)** Presence of a monocyte (Mo) recently infiltrated into the dermis but not yet differentiated into a macrophage.

The ultrastructural analysis revealed keratinocytes in the spinous layer of the epidermis with the presence of keratohyalin granules; however, there was a loss of contact with adjacent keratinocytes since there are regions absent of tonofilaments and desmosomes (Figure 6A). The regions of the epidermis present a necrotic cell with a pyknotic nucleus, and above are cells of the basal layer with mitochondria and absent mitochondrial cristae (Figure 6B). In fibroblasts, the endoplasmic reticulum was presented with dilated cisternae and mitochondria with few cristae (Figure 6C). There was also the presence of dendritic cells with few cristae in mitochondria and intense production of vesicles (Figure 6D). The presence of lymphocytes infiltrated in the dermis between collagen fibers was observed (Figure 6E), in addition to monocytes recently infiltrated in the dermis, not yet differentiated into macrophages (Figure 6F).

## 4 Discussion

CHIKV is considered an arbovirus, transmitted mainly through *Aedes* mosquito bites; hence the skin is the gateway to infection. The virus is known to infect and replicate resident skin cells (skin fibroblasts and dermal macrophages). Next, the virus disseminates to draining lymph nodes and finally is released into the blood circulation to reach several organs, such as the spleen, liver, brain, and muscles (Lum and Ng, 2015). Intense fever and arthralgia are the most evident symptoms of Chikungunya fever; however, dermatological manifestations are also reported in this arboviral infection. Most published studies on CHIKV in the skin are from India (Inamadar et al., 2008; Mavalankar et al., 2007; Passi et al., 2008; Riyaz et al., 2010), which suggest that there is a broad spectrum of cutaneous and mucous manifestations related to the disease. We must take into account the genetic diversity of human populations as well as consider the skin phototype, which is higher in Indian patients. Nevertheless, it was also reported on La Reunion Island (Borgherini et al., 2007), Thailand (Benjamanukul et al., 2022), and Brazil (Turtos et al., 2019).

The cases analyzed here occurred during a Chikungunya epidemic in Campos dos Goytacazes, located in the North Fluminense region of the state of Rio de Janeiro, Brazil. Most of the cases (8/9) were from female patients. According to Salje et al. (2016), female patients are 1.5 times more likely to become infected with CHIKV than male patients. One of the reasons is that women spend more time at home (in and around) compared to men since *Aedes aegypti* is known as a domestic mosquito (Salje et al., 2016; Silva et al., 2023). A systematic review published in 2023 in the Acta Tropica Journal reported that most cases of Chikungunya occur in female patients (ranging from 7.5 to 100.0%) (Silva et al., 2023). In our study, we found more female cases, such as Riyaz et al. (2010). However, in the literature, it is possible to find studies with the predomination of males (Inamadar et al., 2008; Valamparampil et al., 2009) but also with both sexes equally affected in another study (Bandyopadhyay and Ghosh, 2010). The individuals presented erythema, urticated and scaly plaques, and vesiculobullous/vesicopustular lesions. After the lesion biopsy during the acute phase (the samples were collected until 12 days after the onset of symptoms), histology was analyzed as evidence of alterations. Some skin biopsies evidenced acanthosis, which is characterized by an increased thickness of the epidermis due to hyperproliferation of keratinocytes. Disturbed regulation of epidermal proliferation may be caused by mitogenic stimuli such as growth factors and cytokines (Niehues et al., 2022). All nine biopsies showed inflammatory infiltrates around blood vessels and permeating the dermis, also seen in dengue (Thomas et al., 2010) and Chikungunya infections (Riyaz et al., 2010; Sharp et al., 2021). Acanthosis, vacuolar degeneration of the basal cell layer, presence of necrotic keratinocytes, and inflammatory infiltrate were observed also in Zika infection and seem to be due to a direct viral cytopathic effect (Paniz-Mondolfi et al., 2019). Vesiculobullous lesions were also evidenced in a pregnant woman (Benjamanukul et al., 2022), a 48-year-old CHIKV-infected man (Pakran et al., 2011), and small infants (Robin et al., 2010). Dermal edema was also reported during CHIKV infections (Kumar et al., 2017).

CHIKV infects different cells, including epithelial cells, endothelial cells, fibroblasts, and macrophages (da Cunha et al., 2017). Epithelial cells and fibroblasts are among the cells most infected with CHIKV (Tang, 2012). In mouse models, it has been demonstrated that CHIKV has tropism to cells of the deep dermis (Couderc et al., 2008) and dermal fibroblasts (Young et al., 2019). In *in vitro* studies, CHIKV was able to infect culture explants of freshly biopsied human skin (Bryden, 2020) and human dermal fibroblasts (Ekchariyawat et al., 2015). In human skin biopsies, molecular analysis has evidenced the presence of CHIKV RNA (Benjamanukul et al., 2022), and CHIKV antigen was detected by immunohistochemistry in eight of nine cases, with and without a rash (Sharp et al., 2021). In our study, in five out of six cases tested, the presence of CHIKV RNA was observed. RT-qPCR was not performed in all skin specimens due to insufficient material or because tissue collection was not performed. However, we detected CHIKV antigen in all nine cases, mainly diffused in the epidermis and endothelial cells from blood vessels from the dermis and dermal cells, confirming the susceptibility of these cells to infection. In addition, around blood vessels, cells composing the inflammatory infiltrate were also positive, together with sebaceous glands, sweat glands, arrector pili muscle, and hair follicles. Moreover, the susceptibility of all these cells to CHIKV may facilitate the spread of the virus.

In addition to being a physical barrier, the skin is considered an immune organ with several functions (Chu, 2012; Santoro and Boyd, 2021); therefore, when a pathogen enters the skin, an immune response against it is elicited, and it is orchestrated by different cell types, such as keratinocytes, melanocytes, and dermal fibroblasts, in addition to immune cells, such as mast cells, macrophages, and lymphocytes. Together, these cells contribute to expressing cytokines and chemokines that play a vital role in the pathogenesis of cutaneous disorders since they regulate immunity and inflammation. Cytokine and chemokine expression is important to maintain skin homeostasis; however, any imbalance may be detrimental to the resolution of the infection (Hänel et al., 2013; Jiang et al., 2020).

Areas with inflammatory infiltrates of the biopsy skin were characterized by detecting lymphocytes (CD4^+^ and CD8^+^) and macrophages (CD68^+^) cells. These cells were preferentially found in the perivascular areas of the dermis (Kortekaas Krohn et al., 2022). No significant differences were observed between cases and controls for the CD4^+^ cells but for CD8^+^ T cells, which can perform a cytotoxic function or act in the secretion of cytokines that will also activate macrophages (Al Moussawy and Abdelsamed, 2022) and seem to be higher during the acute phase (Wauquier et al., 2011). CHIKV-infected skin exhibited an increased number of macrophages, which are responsible for phagocytosis to destroy the pathogen and avoid its replication (Flannagan et al., 2015). We found expression of pro-inflammatory molecules, such as TNF-*α* and IL-6, mainly in the epidermis, blood vessels, and cells of inflammatory infiltrates. These two were expressed in the Chikungunya cases group with a statistically significant difference when compared to skin controls without infection. TNF-α is the main cytokine regulator in inflammatory diseases, expressed after skin injuries (Banno et al., 2004) and associated with increased endothelial permeability and edema (Royall et al., 1989), while IL-6, which is known as a biomarker associated with severe Chikungunya fever (Ng et al., 2009), is also overexpressed in epidermal layers in cases of barrier disruption of the skin (Bonifati et al., 1994; Hänel et al., 2013), which is commonly observed in psoriasis patients (Johnson et al., 2020). In addition, this cytokine also plays a role in keratinocyte proliferation and leukocyte infiltration (Lin et al., 2003), corroborating our histopathological findings. Inflammatory response in the skin is important in controlling infections; however, the exacerbation of it, with high production of cytokines, may induce immunopathological conditions, leading to cutaneous manifestations, such as erythema, and urticarial and vesiculobullous lesions (Kaya et al., 2020). In addition, RANTES expression was higher in the CHIKV group. This chemokine is an important chemoattractant for eosinophils, T lymphocytes, and monocytes (Kato et al., 2006; Marques et al., 2013) and considered a biomarker associated with severe Chikungunya fever (Ng et al., 2009).

Since it was observed considerable involvement of endothelial cells, we investigated the vascular endothelial growth factor receptor 2 (VEGFR2). VEGF, a vascular endothelial growth factor, is a contributor to angiogenesis, which is an important component of wound healing (Johnson et al., 2020). The binding of VEGF to VEGFR2 is important in physiological processes since it promotes endothelial cell proliferation, increases vascular permeability, and forms new vessels (Holmes et al., 2007). However, it plays a role in pathological conditions, such as inflammation (Shibuya, 2013). In DENV infection, it seems to contribute to vascular permeability (Rattanamahaphoom et al., 2017). The upregulation of VEGFR2 is observed in skin diseases, such as psoriasis (Man et al., 2008). Despite there being no statistically significant difference in the case group compared to the controls, the expression of VEGFR2 was upregulated in some cases studied here.

For a decade, Brazil has been suffering from Chikungunya epidemics, and the virus circulates widely, causing several cases, severe cases, and deaths. Despite that, there are still few studies focusing on dermal changes caused by CHIKV infection, especially considering the immunopathogenesis of the skin. Therefore, in this study, we aimed to fill some gaps in the knowledge about the pathogenesis and clinical course of the disease in this important organ.

## Data Availability

The original contributions presented in the study are included in the article/Supplementary material, further inquiries can be directed to the corresponding author/s.
