# Supplementary material for: Chikungunya virus infection in the skin: histopathology and cutaneous immunological response
Source: Front Microbiol. 2025 Jan 28;16:1497354. doi: 10.3389/fmicb.2025.1497354 (PMC11811090; doi:10.3389/fmicb.2025.1497354)
Supplement: Supplementary file 1 [file Data_Sheet_1.PDF]

Supplementary Material

Table S1. *Epidemiological and clinical information's*

**Sex**	**N/T**	**Symptoms**	**N/T**
Feminine	8/9	Lymphadenomegaly	1/9
Masculine	1/9	Paresthesia	2/9
**Age (years)**	**N/T**	Myalgia	2/9
26-36	3/9	Nausea	3/9
37-47	1/9	Edema	3/9
48-59	5/9	Vomiting	3/9
**Comorbidities**	**N/T**	Anorexia	3/9
Hypertension	3/9	Diarrhea	4/9
Osteoarthritis	1/9	Bitter taste in the mouth	4/9
Depression	1/9	Pruritus	5/9
		Headache	6/9
		Arthralgia	8/9
		Fever	8/9

Table S2. *Skin clinical observations*

**Skin clinical manifestations**	**N/T**
Dryness	1/9
Crusts	1/9
Exulceration	1/9
Hyperchromia	1/9
Painful lesion	1/9
Vesicopustular	1/9
Vesiculobullous	2/9
Cellulite	1/9
Edema	1/9
Morbilliform erythematous rash	2/9
Papular erythematous rash	3/9
Urticaria	2/9
Peeling	2/9
Erythema	4/9

Table S3*. Histopathological findings*

Histopathological findings	(N/T)
Vasocongestion	1/9
Presence of melanophages	3/9
Acanthosis	2/9
Intracorneal vesicle	1/9
Subepidermal lesion	1/9
Fibrin	2/9
Solar elastosis	2/9
Extravasation of red blood cells in the interstitium	1/9
Hyperkeratosis	2/9
Infiltrated near the nervous thread	1/9
Interface dermatitis	3/9
Perianexial infiltrate	2/9
Perivascular inflammatory infiltrate	8/9
Blood capillary ectasia	6/9
Spongiosis	2/9
Interstitial edema	8/9
Endothelial edema	5/9
Basal vacuolation	3/9
Apoptotic keratinocytes	2/9
